# King's College Hospital

**Published:** 1902-05-03

**Authors:** 


### KING'S COLLEGE HOSPITAL.

### SPECIAL APPEAL FOR A RECONSTRUCTION FUND.

Quite appropriately—for not only is he an M.D., as well as a K.C.M.G., but an old student of King's also—Sir John Cockburn presided on Monday night at the annual festival dinner of King's College Hospital at the Hôtel Métropole. In proposing the health of the King he made a felicitous reference to His Majesty's interest in hospital matters. No nobler thought, he said, had ever come from the heart of a king than the idea of founding the Hospital Fund which bore his name; and, moreover, he was now leading the crusade against those twin scourges of the human race, consumption and cancer.

The gathering was a large and brilliant one, 100 or more guests sitting down to dinner. Some of the arrangements were, however, not quite all that could have been desired: representatives of the press were somewhat inconveniently placed, while some of the guests scarcely seemed to realise that it is difficult to take note of the utterances of a speaker some distance off while unsubdued conversation is going on close at hand.

After mentioning the receipt of letters of regret for absence from Lord Brassey and others, the Chairman rose to propose the toast of the evening—"Success to King's College Hospital." He need not tell them, he said, how proud he was, as an old student of the hospital, to occupy the position he filled that night. It was necessary that King's should maintain its rank as a first-class medical school. All other schools were going ahead—King's must retain its lead. They were all proud of the many famous physicians and surgeons who had been connected with it. It was from King's College Hospital that Lord Lister's discoveries, which had revolutionised surgery, had been given to the world; and he scarcely need mention Sir William Fergusson, Dr. Todd, Sir Wm. Priestley, and a host of others who had added lustre to its record, and whose names the world delighted to honour.

#### A Hospital Debt not a Disgrace.

As they all knew, the finances of a great hospital were not like the finances of an individual. If a man spent more than his income it was a disgrace; but to a hospital it might almost be said to be an honour to be in debt, because it proved that it was extending its work. The regular sources of income for that hospital bore no relation to its expenditure; £2,500 was the amount of its settled reliable income, while its expenditure exceeded £20,000, in addition to the enormous amount of skilled service gratuitously rendered by the medical staff. So that £17,500 was needed every year to make up the deficit. And beyond that they wanted additional space for the ear and throat department, for the light and electrical treatment of disease, and for the extension of the out-patient department. King's would not benefit as some hospitals would by the establishment of a teaching university for London, which would enable them to place the space now devoted to physical science at the disposal of the hospital, since these subjects were taught at the College itself, and the hospital would therefore not gain in that way. It was necessary for them to spend something like £75,000 to reorganise and extend the hospital and keep it abreast of the times. And it must be remembered that a great hospital like theirs had a usefulness extending far beyond its district, or the country even. It was Imperial—even world-wide—for no discovery was brought to light by the work carried on here which was not eagerly taken up by the Britains beyond the Seas and by the great American Republic. It was one great function of hospitals, in these times when we were almost losing the meaning of the good old word "neighbour," and when often one did not hear of one's neighbour's illness till one saw the hearse drive up to the door, to help men to do their duty to their neighbour; and in no country in the world was it so well carried out as under that splendid system of voluntary supported hospitals which were the pride of the land. It was a great pleasure to him, after an absence of 27 years, to visit the old place and walk through the breezy old wards. He could not but admire the enormous strides that had been made in many directions, but at the same time it was obvious that a larger amount of good work could be done, with less inconvenience to the patients, if the out-patient department were extended so as to lessen the overcrowding. No better cause than this could come before the public; and he was sure that seed-time and harvest of generous contributions would never fail. He was glad to be able to announce that next year the Lord Chief Justice, Lord Alverstone, would honour the hospital by taking the chair at the annual dinner, and that announcement would, perhaps, help to cover any shortcomings in his own case. He coupled with the toast the name of Lord Dillon.

Viscount Dillon regretted that he could not respond with the energy and brilliance of their friend from across the sea, but he felt sure they would all heartily concur in the happy terms in which the Chairman had described the wants and needs of the hospital. He thanked him on behalf of the committee of management, the medical and surgical officers, and the staff generally for the vigorous terms in which he had set them forth. King's College Hospital had well maintained its position during the past year; the work had been going on steadily, but their wants were great. They could not extend their buildings, since they were hemmed in practically all round, and the County Council would object if they went upward like a Tower of Babel. But much could be done by rebuilding and reorganising, since when the premises were put up over 60 years ago, they were built so that some parts were on a scale out of proportion to the rest. In conclusion he mentioned as a proof of the excellence of the training under their admirable matron, that, of the three civilian ladies chosen for Queen Alexandra's Imperial Military Nursing Corps one was from King's and one from St. Thomas's.

### The Claims of King's College Hospital on Wealthy Churchmen.

The toast of "The Imperial Services" (the Church, the Law, and the Imperial Forces), was proposed by Sir Henry Burdett, whose reference to the fact that King's is regarded as a Church institution called forth some dissent; but who strongly maintained his point that it was to wealthy Churchmen the hospital should look to make its support their first care, and so make it financially as strong as from the work it did it deserved to be.

On rising he said that when he looked at the toast, which, owing to Lord Brassey's absence, he was deputed to propose, he felt like Oliver Twist—inclined to ask for more; for surely if there was one service which was entitled to be called Imperial, it was the medical service. However, that would perhaps come properly within the scope of the next on the list—"The Medical Staff of the Hospital." Dealing first with the Church, he was inclined to think that one reason why that hospital was not financially as sound as it ought to be, considering the results achieved, was because it was recognised as a Church institution. Dr. Wace said, "No, no." But he (Sir Henry) said "Yes, yes," and he would tell them why. The men of the largest means who were doing so much for hospitals generally, said they could not give to any sectarian charity whatever. Of course, King's College Hospital was not sectarian, but they could not get people out of that ridiculous and utterly wrong impression. So he said, be wise in your day and generation. As you are looked upon as a Church institution, go to the Church; drive it home to the minds of wealthy Churchmen that this feeling exists, and let them know that you look to them for special support. If they did that he believed it would not be long before King's College Hospital had a revenue commensurate with its needs and its work. The need for giving personal service as well as money was growing more and more. Out of 4,000 schemes put before her to commemorate her Diamond Jubilee the late Queen selected the cause of the hospitals; and the King became so attached to the scheme that when, in the natural order of things he would have relinquished his connection with it, he said, "No; I can't let it go," and decided that it should be called King Edward's Fund; and that scheme had done immense good to the King and to the whole world. Dealing next with the Law, Sir Henry spoke emphatically of the respect and admiration in which British law and its administration was held all over the world. And as King's was situated in Law-land it was imperative that the representatives of the Law should take it specially in hand. With regard to the Imperial forces, no word of his was wanted to commend them. They had been throughout this great war a wonder to the whole world. If the German army, for instance, had been brought *vis-à-vis* with the new form of warfare he believed its disasters would have been infinitely greater than any that had befallen us. But what would have told in their favour would have been the much sterner measures they would have adopted towards the enemy in the field. He remembered that at the commencement of the war he had to propose the toast of "The Imperial Forces," and he pointed out then that we were the only nation on the earth that imposed on all employers of labour liability for injury to their men; and yet that was not imposed on the Government, the greatest employer of labour, in the most dangerous of occupations—war. But, in a conversation he had recently had with Lord Roberts, he was reminded that every man was now provided for under warrant, and the chances of his going into the workhouse, if he was a proper man, had ceased. He coupled with the toast the names of Dr. Robertson, Mr. Rowden, K.C., and General Stirling.

These gentlemen having responded, Dr. Wace proposed "The Medical Staff of the Hospital," to which Dr. Tirard replied, after which Mr. Awdrey, the treasurer, proposed the Chairman, and in doing so announced that the result of the dinner so far had been the collection of £1,850. He had no doubt there was more to come, and he appealed for it very strongly, since they were working for the poor, for science, and for the health of the nation.

